# Matrix Metalloproteinase-9 Expression by Hodgkin-Reed-Sternberg Cells Is Associated with Reduced Overall Survival in Young Adult Patients with Classical Hodgkin Lymphoma

**DOI:** 10.1371/journal.pone.0074793

**Published:** 2013-09-24

**Authors:** Antonio Hugo Campos, Jose Vassallo, Fernando Augusto Soares

**Affiliations:** 1 Department of Anatomic Pathology, A C Camargo Cancer Care Center, São Paulo, SP, Brazil; 2 Laboratory of Investigative and Molecular Pathology – CIPED, Faculty of Medical Sciences, University of Campinas, Campinas, SP, Brazil; Robert Wood Johnson Medical School, United States of America

## Abstract

Previous studies have investigated the prognostic relevance of MMP9 in classical Hodgkin lymphoma (cHL), with negative results. However, we have found that MMP9 immunoistochemical expression by Hodgkin-Reed-Sternberg cells is associated with reduced overall survival in a subset of young adult Brazilian patients diagnosed with cHL. Additionally, we have observed that MMP9 expression by neoplastic cells in cHL is associated with EBV positivity. These results may support a rational basis for additional studies on the role of this metalloproteinase as a target for therapy in classical Hodgkin lymphoma.

## Introduction

Hodgkin-Reed-Sternberg (HRS) cells of classical Hodgkin lymphoma (cHL) are derived, in most cases, from pre-apoptotic germinal-center B-cells [1]. One of the characteristics of cHL is the abundant inflammatory microenvironment (accounting for over 99% of the tumor mass), which is a result of the production of many chemokines and cytokines produced by HRS cells [2].

Standard therapy regimens currently cure more than 80% of patients diagnosed with cHL. However, some patients will be refractory to therapy and relapse at an early or late phase, requiring salvage therapy and autologous transplant [1],[3]. Consequently, there is an ongoing search to find prognostic factors capable of predicting outcome.

Matrix metalloproteinase-9 (MMP9) belongs to a family of enzymes known to be involved in extracellular matrix degradation and, consequently, processes of invasion and metastasis of many human tumors [4]. Additionally, some studies have suggested that this enzyme may have a role in the regulation of the immune system [5]–[7]. MMP9 expression is also known to be mediated by Epstein-Barr virus infection [8],[9], which is associated with cHL in about 40% of cases [1],[2]. The expression of MMP9 has been related to a poorer prognosis in non-lymphoid tumors and in non-Hodgkin lymphoma [10], but lack of association has been reported in cHL, including a recent Brazilian study with 97 patients [11]–[13].

In this study, we report that MMP9 immunohistochemical expression by HRS cells is associated with reduced overall survival, but not disease-free survival, in adult patients diagnosed with cHL.

## Materials and Methods

### Ethics Approval

This study was approved by the A C Camargo Research Ethics Committee (approval number 753/05), according to institutional and national guidelines. All samples were formalin fixed, paraffin embedded (FFPE) tissues. Written consent was given by the patients for their information to be stored in the hospital database and used for research, as well as the use of left-over biological material. The data was analyzed anonymously. Written consent was obtained from the next of kin, caretakers, or guardians on behalf of the minors/children participants so that their samples and associated data could be stored in research. When written consent was not possible to obtain prospectively, according to national guidelines, the reasons for not doing so were provided to the Institutional Ethics Review Board so that authorization for use of the samples in research could be obtained.

### Case Selection

Paraffin-blocks from 148 retrospective cases of cHL diagnosed between 1970 and 2005 were retrieved from the archives of the Department of Anatomic Pathology of the AC Camargo Cancer Care Center, a tertiary cancer care institution located in the city of Sao Paulo, Brazil. Samples lacking sufficient formalin-fixed and paraffin-embedded tissue to perform immunohistochemical (IHC) analysis, relapse biopsies and HIV-associated HL were excluded. Histological diagnosis was revised with the use of immunostains when necessary. A tissue microarray (TMA) was built as reported elsewhere [14]. Each case was spotted in duplicate. Clinical data were collected from patients’ files and included age, gender, presence of B symptoms, Ann Arbor staging, and, for patients aged over 15 years, the International Performance Status (IPS) [15]. Patients were treated with doxorubicin, bleomycin, vinblastine and dacarbazine ABVD (n = 43), ABVD-equivalent regimens (n = 93) or a combination of bleomycin, etoposide, doxorubicin, cyclophosphamide, vincristine, procarbazine, and prednisone (BEACOPP) (n = 4). Radiotherapy consolidation after chemotherapy was administered to 111 patients. Eight patients with localized disease and favorable prognosis were elected for exclusive radiation therapy.

### Immunohistochemistry and ‘In situ’ Hybridization

Immunohistochemistry was performed manually, as previously described [16], with primary antibodies to MMP9 (polyclonal, 1∶100 dilution, Thermo Scientific, Fremont, CA, USA) and to the EBV latent membrane protein-1 (LMP-1, clone CS1-4, 1∶100 dilution, Novocastra, Newcastle upon Tyne, UK). Cases were also tested for the presence of EBV RNAs using an in situ hybridization (ISH) kit (EBER oligoprobe, Novocastra). A previously known positive case of cHL was used as an external positive control. Negative controls were also used in each run, by omitting the primary antibodies on the same case used as positive control.

After staining, slides were evaluated by three hematopathologists (authors of this study), that were blinded to case details such as age, gender, stage, EBV status or outcome. Only neoplastic cells (classical diagnostic Reed Sternberg cells, Hodgkin cells or variants with undisputable morphology) were considered. Because the number of neoplastic cells varied among cylinders of the same case and between different cases, only the intensity of staining was evaluated for MMP9. Staining was graded between + (weak staining) and +++ (strong staining), as previously reported [16]. The authors evaluated the slides separately, and cases in which there was disagreement with regard to intensity of staining were evaluated by the three authors together in order to reach a consensus. As weak staining might have a questionable biological meaning, cases were considered either negative (absent or weak staining) or positive (moderate or strong staining) for statistical purposes. EBV was considered positive or negative for LMP1 and/or EBER following the recommendations by Gulley et al [17]. Images were obtained using the Aperio ScanScope XT Slide Scanner (Aperio Technologies, Vista, CA, USA) and the Aperio ImageScope Software (version 10.2.2.2317, Aperio Technologies, Vista, CA, USA).

### Statistical Analysis

Statistical analyses were performed using the statistical package Graph Pad Prism (version 5.02, GraphPad Software Inc., San Diego, CA, USA) or MedCalc software (version 11.0.1, MedCalc Software, Belgium). Pearson chi-square or Fisher’s exact tests were used to assess the relationship between MMP9 expression, EBV status and other clinic-pathological variables. Differences were considered statistically significant for *P*<0.05. *Post hoc* power analyses were performed using G*Power Software (version 3.1.7) [18].

Overall survival (OS), defined as the time between diagnosis and the date of death (by any cause), and disease-free survival (DFS), defined as the time elapsed from end of treatment to recurrence, were determined by the Kaplan-Meier method. Other end-points (such as progression free survival) or response criteria (such as partial or complete response) could not be reliably calculated or obtained from the clinical records, according to standard guidelines [19]. Statistical associations between individual variables, OS and DFS were analyzed using the log-rank test. For survival analysis, patients were divided into subgroups (up to 15 years of age, between 15 and 45 years, and above 46 years). *Post hoc* power analyses of primary outcomes were performed using Graph Pad StatMate (Version 2.00, GraphPad Software Inc., San Diego, CA, USA). Individual variables with a *P* value less than 0.2 in univariate analysis were tested in a multivariate analysis, using the Cox proportional hazards model in a backward stepwise selection, as previously reported [20].

## Results

### MMP9 Expression by HRS Cells and its Association with Individual Variables

**Figure 1:**
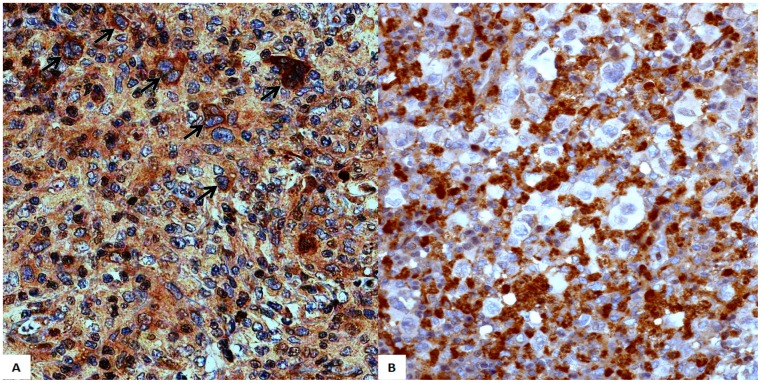
MMP9 expression in classical Hodgkin lymphoma (200x). (A) Cytoplasmic expression of MMP9 by Hodgkin-Reed-Sternberg cells (arrows). Variable staining is also observed in cells of the reactive background. (B) A case with positive cells in the reactive background, but no expression of MMP9 in Hodgkin-Reed-Sternberg cells.

From the original 148 cases included in this study, 118 presented satisfactory material and information to be included for analysis. An analysis of the clinical characteristics and outcome of both groups (118 patients in which MMP9 expression by HRS cells could be assessed *versus* 30 patients in which there was no material on the TMA slides) showed no significant differences with regard to EBV frequency, age distribution, gender, histology, Ann Arbor stage, B symptons, IPS status or outcome. The main clinical and pathological features of the remaining 118 cases are listed on **Table 1**. MMP9 expression was observed in the cytoplasm of neoplastic cells in 77 out of 118 patients (65.2%, **figure 1**). MMP9 expression was also detected with variable intensity in the reactive background. Fifty-five out of 118 cases (46.6%) were associated with EBV infection by combined expression of LMP-1 and EBER1. We observed an association of MMP9 expression by neoplastic cells with EBV infection, but not with other individual variables (**table 2** **)**. The power analysis showed that the present sample size had a power of 99%, 46%, 85%, 7%, 4% and 5% to detect an association of MMP9 expression by HRS cells with age, gender, histology, Ann Arbor staging, IPS and B symptons, respectively.

**Table 1:** Clinico-pathological features of 118* cHL patients.

Parameter	No.	%
**EBV status**		
Positive	55	46.6
Negative	63	53.4
**Age (years)**		
<15	29	24.6
15–45	74	62.7
>45	15	12.7
**Gender**		
Male	62	52.5
Female	56	47.5
**Histology**		
Nodular sclerosis	77	65.3
Mixed celularity	30	25.4
Lymphocyte rich	6	5.1
Lymphocyte depletion	1	0.8
Unclassifiable	4	3.4
**Ann Arbor Staging**		
I	12	10.2
II	42	35.6
III	36	30.5
IV	21	17.8
NA	7	5.9
IPS*		
0–2	58	65.2
>2	19	21.3
NA	12	13.5
**“B” symptons**		
Yes	47	39.8
No	68	57.6
NA	3	2.5
**Follow-up**		
ACR	82	69.5
AWD	8	6.8
DOD	24	20.3
DOC	3	2.5
LFU	1	0.8

IPS - International Prognostic Score; NA - data not available; ACR – alive in complete remission; AWD - alive with disease; DOD - dead of disease; DOC - dead of other cause; LFU - lost to follow up; (*) For IPS score, data are reported only for patients aged over 15 years (n = 89).

**Table 2:** Correlation of MMP9 expression with EBV status and clinico-pathological features.

Parameter	MMP-9 NEG	MMP-9 POS	*P*
**EBV status**			
Positive	14	41	**0.03**
Negative	28	35	
**Age (years)**			
<15	5	20	0.18
15–45	31	47	
>45	6	9	
**Gender**			
Male	17	45	0.05
Female	25	31	
**Histology**			
Nodular sclerosis	28	49	0.56*
Mixed celularity	8	22	
Lymphocyte rich	2	4	
Lymphocyte depletion	1	0	
**Ann Arbor Staging**			
I–II	18	36	0.69
III–IV	22	35	
IPS**			
0–2	28	46	1.00
>2	10	18	
**“B” symptons**			
Yes	27	47	0.70
No	19	28	

POS - positive; NEG - negative.

Fisher’s exact test or Qui-square test.

(*) For statistical purposes, the categories “lymphocyte rich” and “lymphocyte depleted” have been grouped together; (**) data reported only for patients aged over 15 years.

### Prognostic Analysis

**Figure 2:**
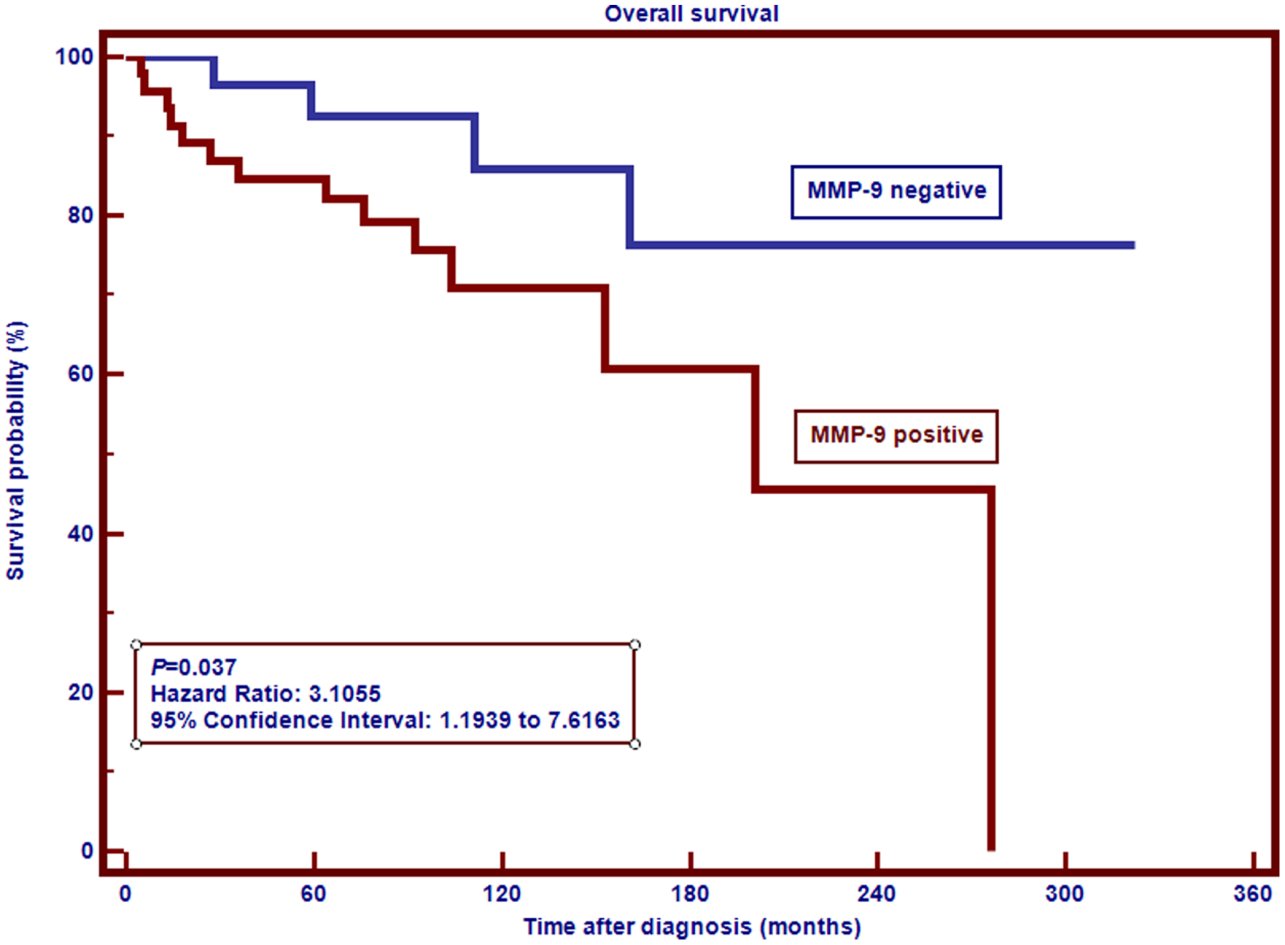
Overall survival probability of 78 patients diagnosed with classical Hodgkin lymphoma between 15 and 45 years of age according to MMP9 expression.

A shorter OS was associated with IPS>2 (*P*<0.0001), Hemoglobin level<10.5 (*P = *0.0006), Lymphocyte count (*P = *0.02), Ann Arbor stage (*P = *0.03), and with the expression of MMP9 by neoplastic cells (*P = *0.03, **figure 2**). In the prognostic analysis of MM9 expression by HRS cells, the survival difference between MMP9-positive group and MMP9-negative group was 0.308 (30.8%) and the analysis had between 95% and 99% power to detect a survival difference of 0.308 with a significance level (alpha) of 0.05 (two-tailed). The only factors associated with reduced disease-free survival were IPS>2 (P = 0.0009) and Hemoglobin level<10.5 (P = 0.01). Only patients between 15 and 45 years of age were included in the analysis. Pediatric patients and patients aged over 45 years were not assessed because insufficient numbers of cases were available in these subgroups for meaningful statistical analysis.

In the multivariate analysis (**table 3**), MMP9 expression by HRS cells resisted as an independent predictor of worse overall survival for patients between 15 and 45 years, along with IPS>2 and Hemoglobin levels<10.5. Patients with MMP9 expression by HRS cells were approximately 10.3 times more likely to die than those with no MMP9 expression by HRS cells.

**Table 3:** Multivariate Cox Regression Analysis for Overall Survival of patients with classical Hodgkin lymphoma between 15 and 45 years of age (n = 64).

Covariates	HR	95% CI	*P*
MMP9 expression (positiveversus negative)	10.3335	2.0219 to 52.8135	0.0052
Hemoglobin level<10.5 g/dl	8.4695	1.6028 to 44.7546	0.0123
IPS>2	7.6957	1.6556 to 35.7718	0.0096

HR: Hazard Ratio; CI: Confidence Interval; IPS: International Prognostic Score.

## Discussion

We report that MMP9 expression by HRS cells is independently associated with an adverse outcome in patients with cHL between 15 and 45 years. Moreover, MMP9 expression by neoplastic cells appears to be an independent prognostic predictor of worse overall prognosis for this subgroup of patients. There was no influence of MMP9 expression by neoplastic cells on disease-free survival.

The expression of MMP9 has been associated with increased aggressiveness and poor prognosis in patients with non-Hodgkin lymphoma [10], but an influence on prognosis has not been reported in the few studies on cHL [11]–[13]. Flavell et al. [11] reported that the survival analysis performed on their cases was compromised by the fact that there were a small number of patients in the MMP9-positive group, although there was a tendency toward an adverse outcome in this category. In the study conducted by Kuittinen et al. [12], MMP9 expression by neoplastic cells also showed a tendency toward an adverse outcome. However, the antibodies, dilution and immunohistochemical techniques in both studies were different from those used by our group.

The MMP9 antibody used in our study was the same used by Souza et al. [13] in their study with 97 Brazilian patients, although the dilution was different (1∶100 *versus* 1∶200). Additionally, differences in immunohistochemical staining evaluation and the use of TMA in our study might have accounted for the discrepant results regarding the prognostic relevance of MMP9. Souza et al. [13] analyzed patients over 18 years old. Using the same approach, we also did not observe influence of MMP9 expression by neoplastic cells in cHL on overall survival, although there was a trend toward worse prognosis (P = 0.0681, Hazard ratio = 2.1745, 95% CI = 0.9901 to 4.7757). When we decided to divide patients by age-defined groups (pediatric up to 15 years of age, young adults between 15 and 45 years and adults over 45 years), the prognostic relevance of MMP9 expression in the subgroup of patients became apparent. Moreover, MMP9 expression by neoplastic cells remained as an independent prognostic factor of overall survival. Stratification by age has been increasingly used in studies involving patients with Hodgkin lymphoma (HL), since constitutional differences, particularly the state of the immune system (not fully developed in children and young adolescents and not so effective in the elderly) appear to influence the development of the disease and the effect of factors such as EBV infection and expression of CD20 [21]–[23].

Our study, however, has some limitations. One of them is the inclusion of patients treated with ABVD or ABVD-equivalent regimens, with a small number of patients treated with BEACOPP, although this approach has already been used in other studies [3],[13],[24]. Additionally, bias in statistical analysis, such as “overfitting” [25], could have existed. To minimize this, a careful approach was chosen in the multivariate analysis and the number of predictors was relatively small. Furthermore, the demographical features of the patients, and the fact that the classical prognostic features were maintained, speak in favor of the representativeness of our setting. It may also be possible that the lack of association of MMP9 expression by HRS cells with DFS may have been biased by limitations inherent to this kind of endpoint (based in tumor assessment that, if not precisely noted in medical records, may have resulted in inaccurate recording of disease recurrences or censored events). Finally, this cohort of patients displays an overall survival rate of 78%, which is lower than expected even among high risk patients. This may be due to social inequality, which is high in Brazil and have been shown to influence treatment response and survival rates in a previous study with Brazilian patients [26]. Thus, a large cohort of patients between 15 and 45 years of age homogeneously treated is needed to verify if MMP-9 expression by HRS cells could indeed be used as an independent prognostic factor. Likewise, studies with a larger population of patients below 15 years and over 45 years of age are needed to investigate the prognostic importance of the expression MMP-9 expression by HRS cells in these subgroups of patients.

Our finding that MMP9 was associated with EBV-infection is in line with functional studies that reported that MMP9 expression is enhanced by LMP1 [8],[9]. However, these studies used cell lines derived from Burkitt lymphoma and cervical carcinoma. Other immunoistochemical studies investigating the association of MMP9 with EBV infection in cHL have reported negative results [11],[13], and in our study MMP9 status did not correlate with Mixed Cellularity subtype (more frequently associated with EBV infection). Nevertheless, it has been already published that in Brazil, as in other developing countries, Nodular Sclerosis subtype association with EBV may be higher [27]. To clarify if MMP9 expression by neoplastic cells is more commonly associated with EBV-infection regardless of histological type, our finding need to be confirmed by functional studies using HL-derived cell lines.

The exact role of MMP9 in the biology of cHL also has to be investigated. Although MMP9 is commonly known for its role in the degradation of extracellular matrix [4], some reports have suggested that the expression of MMP9 by neoplastic cells is associated with immune system processes [5],[6]. The reports that MMP9 may be associated with immune tolerance are particularly interesting, since modulation of the immune system by HRS cells towards a tollerogenic state is well described [1],[2]. Additionally, the expression of MMP9 by human promyelocytic leukemia HL-60 cell line has been associated with intercellular adhesion molecule-1 (ICAM-1) shedding into the cell medium [28], which would suggest another explanation. ICAM-1, which is overexpressed by HRS cells [29], regulates leukocyte homing, activation, and effector functions like T-cell and Natural Killer (NK) cell-mediated cytotoxicity. ICAM-1 shedding by tumor cells into the microenvironment might be associated with T/NK-cell mediated death resistance, since soluble ICAM-1 would act as a decoy ligand, binding surface receptors of NK-cells and preventing them from recognizing molecules displayed on the surface of the tumor cells. In fact, ICAM-1 is detected at high levels in the serum of patients with HL, and has been associated with significantly lower survival in HL [30],[31].

In conclusion, MMP9 expression by HRS cells is associated with reduced overall survival in patients between 15 and 45 years of age and appears to be an independent prognosis of survival for this subgroup. Since antitumor therapy based on the inhibition of metalloproteinase is still evolving [32],[33], the expression of MMP9 by HRS cells might be used to identify patients who are at higher risk of unfavorable outcome, and therefore represent potentially eligible individuals for this therapy.
